# Health-Related Quality of Life in Psoriasis: Literature Review

**DOI:** 10.3390/jcm13164623

**Published:** 2024-08-07

**Authors:** Gioele Ghezzi, Antonio Costanzo, Riccardo G. Borroni

**Affiliations:** 1 Humanitas University, 20072 Pieve Emanuele, MI, Italy; gioele.ghezzi@humanitas.it; 2 Dermatology Unit, Humanitas Research Hospital—IRCCS, 20089 Rozzano, MI, Italy; antonio.costanzo@hunimed.eu; 3 Department of Biomedical Sciences, Humanitas University, 20072 Pieve Emanuele, MI, Italy

**Keywords:** quality of life, outcome measure, psoriasis, questionnaire

## Abstract

The assessment of quality of life (QoL) in patients with psoriasis plays a crucial role in understanding the impact of the disease and evaluating treatment outcomes. We provide an overview of the key measures used to assess QoL in psoriasis patients, including both generic and psoriasis-specific instruments. The limitations and strengths of instruments such as the Dermatology Life Quality Index (DLQI), Skindex, and Psoriasis Disability Index (PDI) are discussed, highlighting their psychometric properties and areas for improvement. Furthermore, this review examines the potential of disease-specific QoL measures in providing greater sensitivity to disease-related burden and change compared to generic instruments. However, most of the available psoriasis-specific patient-reported outcome measures need further validation. We aim to provide valuable insights into the importance of using validated QoL measures in clinical practice and research, ultimately contributing to a more comprehensive assessment of the impact of psoriasis on patients’ lives and enhancing the evaluation of treatment interventions.

## 1. Introduction

The skin is the most visible organ on the body’s surface and acts as a sensory organ, providing information about pain, pleasure, temperature, and pressure. Furthermore, the skin is where important events and processes that are essential for our thoughts, emotions, and social interactions take place [1]. It has been theorized that the skin is a crucial component of the mind’s structures and functions [2]. The World Health Organization (WHO) defines quality of life (QoL) as “an individual’s perception of their position in life in the context of the culture and value systems in which they live and in relation to their goals, expectations, standards and concerns” [3]. Skin changes have a significant impact on how we view ourselves and how others perceive us, as well as our overall physical and mental health. Skin conditions are collectively ranked as the fourth most common cause of non-fatal disease burden worldwide [4]. Healthcare providers and scientists have long recognized the influence of skin conditions on various facets of patients’ lives and the potential for successful treatment to enhance patients’ quality of life. Health-related quality of life (HRQoL) is a complex and multidimensional construct that reflects an individual’s quality of life as it relates to their health or disease status [5,6].

**Figure 1:**
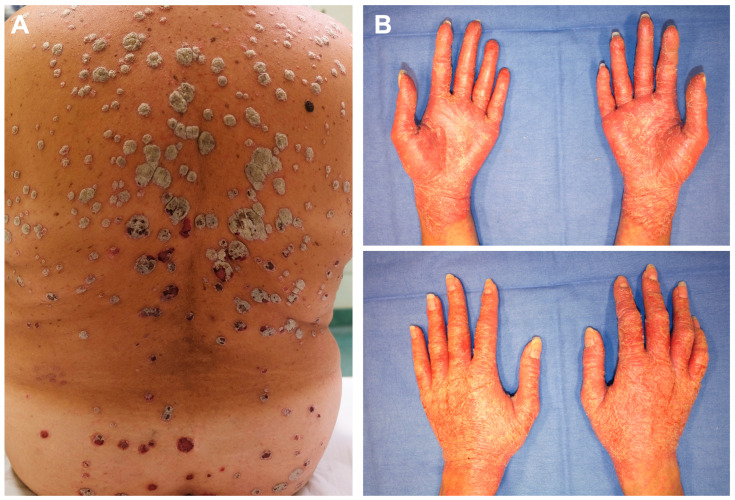
These two women affected by chronic plaque psoriasis (**A**,**B**, respectively) have different extents of body surface area (BSA) affected by skin lesions (**A**, 18 and **B**, 4). However, the impact of psoriasis on quality of life is comparable (DLQI 21) because of different anatomical localization involving the hands in patient (**B**).

Psoriasis, a chronic, inflammatory disease of the skin and joints affecting about 3% of the global population, is associated with disability levels comparable to those of other significant conditions, such as cancer, hypertension, arthritis, diabetes, and heart disease [6,7]. The lesions of psoriasis can cause itching [8,9], pain, and soreness [9,10,11], and in certain instances, the skin may fissure and bleed [8,9]. In addition, individuals with psoriasis experience remarkable psychological distress such as social embarrassment, reduced body satisfaction, anxiety, and depression [8,10,12,13,14,15,16,17], including increased likelihood of suicidal behaviors [18]. Patients must actively manage their psoriasis; however, excessive psychological distress can disrupt effective self-management [19]. Topical treatments are the mainstay of the majority of patients, but the time required for treatment is associated with reduced QoL [20]. Topical treatments’ smell, formulation, or cosmetics and their side effects such as itching, burning, and dryness also hamper their use, eventually leading to dissatisfaction [21]. The literature indicates that individuals suffering from psoriasis might adopt unhealthy coping mechanisms [22], such as excessive alcohol consumption [13,23], overeating [24], and smoking [24,25]. Interestingly, a study showed that significantly more patients with visible lesions have a drinking history compared to those without visible lesions; those with exposed lesions also have a significantly higher percentage of smoking history [26]. Impairment of HRQoL correlates not only with psoriasis severity as measured by clinician-reported outcomes (CROs) but may also be influenced by younger age, female gender [27], scalp or nail involvement, skin lesions on visible areas such as head/neck, and involvement of genitals/groin or hands and feet [28,29,30,31,32] (Figure 1).

Healthcare choices and research on health services rely on standards assessing disease severity and treatment course [33]. The Psoriasis Area Severity Index (PASI) is a clinician-reported outcome (CRO) measure that calculates the skin area extent and lesion appearance (score range, 0–72, with higher scores indicating more severe disease). Body surface area (BSA) can be classified as less than 3% for mild psoriasis, 3% to 10% for moderate disease, and 11% or greater for severe disease. PASI and BSA are the most commonly used CRO measures in psoriasis [34]. The definition of severe psoriasis is important in clinical practice not only for adequate management but also for reimbursement by healthcare providers. In addition to objective evaluation, the severity of psoriasis can be assessed by its impact on social life, self-perception, and physical discomfort. These parameters may all be evaluated by QoL questionnaires. Health-related QoL outcomes are relevant in psoriasis for therapeutic decision making, particularly for systemic therapies [28,35,36]. Remarkably, clinicians often differ with patients’ perceptions on HRQoL. For this, careful and close patient–physician communication is needed to identify fields of risk for QoL impairment efficiently. A literature search was performed through the PubMed database using the keywords “psoriasis”, “questionnaire”, “quality of life”, and “patient reported outcome”. Age (<18), language (only articles written in English were included), psoriatic arthritis, and psoriasis types other than chronic plaque psoriasis were exclusion criteria. In order to find relevant studies, the articles were first assessed, and additional pertinent research was discovered by examining the citations referenced in each included article. This review aims to summarize the current literature on the most validated tools available in English for assessing HRQoL and disease-related QoL in adult patients with psoriasis vulgaris (chronic plaque psoriasis) and discuss future directions for efficiently implementing these tools into clinical practice and research.

## 2. Generic and Dermatology-Specific Health-Related Quality of Life (HRQoL) Measures

Patient-reported outcome (PRO) measures offer patients and healthcare providers a means to evaluate physical and mental functioning, aid in treatment decision making, recognize coping strategies from the patient’s viewpoint in clinical assessments [37,38,39], and enhance communication between patient and clinician [38,40]. Among the generic HRQoL questionnaires, the Short Form 36-Item Health Survey (SF-36) has cross-cultural validation [41] and is widely used in clinical trials. Since its focus consists essentially in the physical dimension, it is inadequate for assessing the psychological sphere of patients. Therefore, it might be useful to associate SF-36 with other tools.

In 1972, Dahl and Comaish stated that psoriasis treatment was “good” if it reduced the extent of lesions or scaling, leading to a noticeable improvement in the patient’s social or professional life [42]. In 1994, Finlay and Khan created the Dermatology Life Quality Index (DLQI) [43], the first tool specifically designed for assessing quality of life in dermatology. This development enabled numerous studies on the effects of skin conditions on patients’ quality of life [44]. The DLQI comprises ten inquiries that delve into various aspects of a patient’s life, including physical functioning, well-being, and social functioning. DLQI scores range from 0 to 30, whereby 0–1 = “no effect”; 2–5 = “small effect”; 6–10 = “moderate effect”; 11–20 = “very large effect”; 21–30 = “extremely large effect”. The DLQI gathers particular details not covered by general HRQoL tools like the SF-36, supporting the necessity for dermatology-focused surveys. The DLQI provides a succinct measurement of certain facets of patient functioning and is generally sensitive to changes at different stages of this disease [45]. A change of four points in the DLQI correlates with the minimum clinically meaningful change in a person’s HRQoL, defined as the smallest difference in the score of a domain of interest that patients consider as beneficial and which would justify a change in patients’ management, in the absence of worrisome side effects and excessive cost [46]. Indeed, DLQI is also an instrument to evaluate treatment success, with treatment goals currently set for DLQI < 2 [47]. The DLQI has been extensively validated, owing to its simplicity and brevity [48]. It has emerged as the most frequently utilized PRO tool in dermatology, with translations available in various languages. Both PASI and DLQI reflect disease burden [49] and predict socioeconomic stress [50]. The correlation between absolute PASI and absolute DLQI, however, is weak [51,52], although there seems to be a correlation between an improvement in PASI and an improvement in the DLQI [53]. Studies reported female gender and higher PASI score as factors impacting DLQI negatively [54,55,56], with younger age showing an association with lower quality of life [56,57]. Disease duration was also linked to quality of life, with the highest HRQoL impairment in patients with long-standing disease [26,54,57].

These instruments are crucial for determining treatment objectives for psoriasis. Along with PASI, DLQI has also been used for outcomes measurement in clinical trials [58,59], patient registries [60,61,62,63], and real-world observations [53,54] as well as in health services research on the quality of care [51,64,65,66]. The impact on HRQoL as measured by DLQI is taken into account also in the definition of psoriasis severity. Following Finlay’s “rule of tens”, severe psoriasis is defined as either PASI > 10 OR BSA > 10 OR DLQI > 10 [67]. The 2011 European consensus definition of moderate-to-severe disease is “(PASI > 10 or BSA > 10) AND DLQI > 10” [68], but this approach might exclude from systemic treatment up to 5.9% of patients who exclusively have a subjective feeling of severity (DLQI > 10 but PASI < 10). In addition, according to the same criteria, treatment efficiency could be somewhat overestimated in 4.1% of patients under systemic therapy whose DLQI is >10, albeit with PASI < 10 [69]. A more recent definition of severe psoriasis is >10% BSA or special areas affected or BSA 5–10% and DLQI > 10 [30]. Higher PASI responders tend to achieve greater improvement in DLQI [70,71], and some studies [72,73] have shown a correlation between changes in PASI and improvements in DLQI.

While its classical psychometric properties (including test–retest reliability, internal consistency, and construct validity) have generally been found to be adequate, more in-depth studies using Rasch analysis (see below) have highlighted that using the DLQI as a unidimensional instrument may not be acceptable, since its total score might not reflect the different domains explored by the questionnaire [74,75]. In addition, the item responses of more than half of the questions are affected by external factors such as age, gender, diagnosis [74,75,76], and nationality [76,77], not solely by the level of HRQoL impairment. This might have implications when using the DLQI to assess the impact of psoriasis in heterogeneous patient populations, or as an outcome measure in large international clinical trials, or in older adults, in which QoL has different focuses than middle-aged adults. Even in the case of equal total QoL scores in patients from different countries, the difference between separate QoL item scores may be significant [78].

The Skindex-29 questionnaire comprises 29 questions examining three areas: symptoms, emotions, and function. It produces domain-specific scores ranging from 0 to 100, with higher scores indicating a more significant impact of skin disease on QoL and functioning. Various studies have validated its applicability to common skin disorders, making it a valuable tool for establishing treatment baseline and assessing QoL [79]. When the Rasch model was initially applied to the Skindex-29, it was discovered that the 29 items did not conform to the model. After 12 items were removed and the response categories were regrouped, resulting in two scales instead of three, the Skindex-17, as it was renamed, finally conformed to the model [76]. The first scale examines a subject’s psychosocial functioning, while the second addresses symptoms [80]. The Skindex-17 exhibits significantly less item bias related to age, gender, diagnosis, and nationality, affirming the feasibility of attaining these desirable measurement properties [76,77]. This refinement process was crucial in developing the Skindex-17 and underscores the commitment to creating a robust and accurate tool for assessing QoL in patients with skin diseases. The Skindex-16, a versatile tool derived from the Skindex-29, is one of the most widely used dermatology-specific HRQoL questionnaires [81]. In a comparative study, it was found that the Skindex-16 is more sensitive than DLQI in identifying mild impairment in HRQoL [82].

## 3. Psoriasis-Specific Health-Related Quality of Life (HRQoL) Measures

The potential of disease-specific QoL measures is promising, as they can provide greater sensitivity to disease-related burden and change compared to generic instruments [83]. Therefore, they enable the detection of small changes in psoriasis-specific QoL and are suitable both in daily practice and in clinical trials. In addition, they could be helpful in identifying what aspects of psoriasis-related QoL are most important to individual patients. Recently, the measurement properties of some dermatology-specific and psoriasis-specific HRQoL measures used in dermatology have been evaluated according to the Consensus-based Standards for the Selection of Health Measurement Instruments (COSMIN) criteria, which are the current “gold-standard” [84,85]. We review here the most relevant self-reported psoriasis-specific HRQoL measures available in English for psoriasis vulgaris (chronic plaque psoriasis). Data are summarized in Table 1.

### 3.1. Psoriasis Disability Index

One of the first and the most studied PROs specific to psoriasis vulgaris is the Psoriasis Disability Index (PDI), developed by Finlay and Kelly in 1987. The PDI comprises 15 questions that focus on assessing the QoL of adult psoriatic patients [86]. There are two possible scoring systems (visual analog scale from 1 to 7 or tick box with questions answered as either 0 (“not at all”), 1 (“a little”), 2 (“a lot”), or 3 (“very much”). It measures the impact of psoriasis on various aspects of a patient’s life, including daily activities, leisure, occupational/school functioning, and relationships. While the PDI demonstrates psychometric strength as validated by several studies [41], it does not evaluate emotional or psychological well-being in relation to psoriasis, being rather focused on disability. This limitation may hinder its ability to provide a comprehensive assessment of the impact of psoriasis in clinical practice unless it is used alongside an additional measure. Additionally, the PDI has been shown to have poor reproducibility [41], making it unsuitable for clinical trials. Although it exhibits good construct validity, content validity, internal consistency, respondent burden (acceptability), and responsiveness [87], its dimensionality and differential item functioning are poor [41].

### 3.2. Psoriasis Life Stress Inventory

The Psoriasis Life Stress Inventory (PLSI) [88] is a 15-item measure that evaluates the effect of daily stressors, including psoriasis-related experiences and the degree of associated stress, aimed at measuring the social impact of psoriasis. Each item is scored on a four-point scale, ranging from 0 (“not at all”) to 1 (“slight degree”), 2 (“moderate degree”), and 3 (“a great deal”). The total score varies from 0 to 45, with higher scores indicating greater levels of daily stress [88]. There is not sufficient published evidence to determine full face and content validity of the PLSI. Structural validity was rated insufficient [87]. The PLSI has a high degree of internal consistency. It demonstrates comparability with PASI scores. Specifically, patients with a PLSI score of 10 or greater have been found to have greater overall psoriasis severity.

### 3.3. Salford Psoriasis Index

The Salford Psoriasis Index (SPI) [89] is made up of three individually scored measures: (1) signs, (2) psychosocial disability, and (3) intervention. The signs measure converts the PASI score into a number ranging from 0 to 10. The psychosocial impact measure evaluates the effect of psoriasis on daily life, using a visual analog scale of 0 (“not at all affected”) to 10 (“completely affected”). The intervention measure reflects historical disease severity, where extra points are given for the need for systemic treatment, admission to the hospital, and number of episodes of erythroderma [89]. The psychosocial impact measure is strongly associated with the PDI, but it is poorly correlated with PASI [89]. The current psychosocial impact components of the Salford Psoriasis Index showed significant correlation with DLQI [89].

### 3.4. Self-Assessed Simplified Psoriasis Index

The Self-Assessed Simplified Psoriasis Index (SaSPI) is a 13-item measure of psoriasis symptom severity that allows patients to document the areas of their body affected by psoriasis. The SaSPI includes a single item to assess psychosocial impact using a 0–10 visual analog scale; this may not capture the full impact of psoriasis on the emotional well-being and daily functioning of patients. The SaSPI is based on the SPI [89]; however, there is no published evidence of qualitative research to support the content of the SaSPI. Construct validity was demonstrated through a strong correlation with the DLQI, and adequate reliability was also shown [90]. The three SaSPI components include separate indicators of current severity, psychosocial impact, and historical course.

### 3.5. Psoriasis Quality of Life 12-Items

The Psoriasis Quality of Life 12-items (PQoL-12) is a part of the Koo–Menter Psoriasis Instrument (KMPI) [91,92], which was originally developed to assist dermatology providers in the indication of systemic (including biologic) therapies for reimbursement purposes [93]. The PQoL-12 consists of 12 items completed by the patient before the physical examination. The score ranges between 0 (best QoL score) and 120 (worst QoL score), with a statistically significant cut-off threshold separating patients with substantial QoL impairment set to a score of 50 points. The PQoL-12 instrument is valid and reliable and was found to be predictive of PASI [91,92].

### 3.6. Psoriasis Symptom Inventory

The Psoriasis Symptom Inventory (PSI) [94] is an eight-item measure that assesses signs and symptoms of itching, redness, scaling, burning, stinging, cracking, flaking, and pain. The severity of each item/sign or symptom is scored on a scale of 0 (“not at all”) to 4 (“very severe”); the eight items are summed for a total score (range 0–32). The psychometric properties of both the 7-day and the 24 h recall versions [95,96] were tested in patients with moderate-to-severe psoriasis, and construct validity was determined through correlations with the DLQI and SF-36 [96]. The PSI showed evidence of adequate reliability [96] and an ability to detect improvement [97], although there is no published data regarding its responsiveness to deterioration.

### 3.7. Impact of Psoriasis Questionnaire

The Impact of Psoriasis Questionnaire (IPSO) is a questionnaire used to measure the impact of psoriasis, consisting of 16 items with clear scoring and completion instructions [98]. Its reliability has been well demonstrated through various techniques, such as classical test theory and Rasch analysis [87]. However, the four-week recall period may not be the most effective for patient recall and detecting frequent changes. Despite this, both the reproducibility and item bias of the IPSO have been deemed good [41]. A recent systematic review indicated that only the IPSO-11 Rasch version [87] had sufficient evidence for a strong recommendation for use according to the COSMIN guidelines [84,85,99].

### 3.8. Psoriasis Index of Quality of Life

The Psoriasis Index of Quality of Life (PSORIQoL) [100] is a 25-item questionnaire developed from the responses of 62 patients with psoriasis in the United Kingdom, Italy, and The Netherlands that evaluates the impact of psoriasis on quality of life, covering social difficulties, embarrassment, and limitations in daily functioning. The PSORIQoL includes items related to fear of negative reactions from others, self-consciousness and poor self-confidence, problems with socialization, physical contact and intimacy, limitations on personal freedom and impaired relaxation, and sleep and emotional stability. It uses a yes/no response scale, which may not capture smaller changes important to patients and clinicians. Patients receive one point for each item for every negative statement with which they agree. The final scores range from 0 to 25, with higher scores indicating worse psychosocial impact. The questionnaire has shown good psychometric properties, including reliability and validity by Rasch analysis [101]. The PSORIQoL construct validity and content validity, unidimensionality, and reproducibility are good, and respondent burden is acceptable [41]. Of note, the internal consistency of the PSORIQoL is higher than that of DLQI. The PSORIQoL is also thought to be a useful tool in clinical trials [102].

### 3.9. Pictorial Representation of Illness and Self Measure

The Pictorial Representation of Illness and Self Measure (PRISM) [103] is a visual tool consisting of a yellow circular disk and a smaller red disk to represent the respondent’s life and psoriasis, respectively. The patient is asked “Where would you put the illness in your life at this moment?” The main outcome measure is the distance between the two circles, i.e., the self-illness separation score. The PRISM task has been tested in patients with psoriasis but requires a trained administrator and detailed discussion to identify particular difficulties. The resources required mean it is unlikely feasible for routine clinical use.

**Table 1:** Summary of the main features of selected psoriasis-specific HRQoL PRO measures. Modified from [87].

Patient-Reported Outcome Measure	Construct	Recall Period	(Sub)scale(s) (Number of Items)	Response Options	Score Range	Limits	Advantages
Psoriasis Disability Index (PDI) [86]	Psoriasis disability	4 weeks	Daily activities (5), work or school (3), if not at work/school (3), personal relationships (2), leisure (4), treatment (1)	4-Point adjectival scale	0–45	Does not evaluate emotional or psychological well-being, it requires use alongside additional measures, poor reproducibility	Good psychometric properties, except for differential item functioning and dimensionality
Psoriasis Life Stress Inventory (PLSI) [88]	Psoriasis-related stress	4 weeks	1 scale (15)	4-Point adjectival scale	0–45	No sufficient published evidence to determine content validity, insufficient structural validity	High internal degree of internal consistency, comparability with PASI scores
Salford Psoriasis Index (SPI) [89]	Signs, psychosocial disability, and treatment history	The whole disease history of the patient	Signs, psychological disability, and interventions	10-point score for each subscale	0–30	Poor correlation of the psychosocial impact measure with PASI	Good correlation with PDI, holistic approach
Psoriasis Quality of Life 12-items (PQoL-12) [91,92]	Psoriasis-related QoL and symptoms	4 weeks	Quality of life (8) and symptoms (4)	10-point scale	0–120	Only partial correlation with PASI	Validity and reliability
Self-Assessed Simplified Psoriasis Index (SaSPI) [89]	Psoriasis signs and symptoms severity	The whole disease history of the patient	Current severity, psychosocial impact, and historical course	50-point scale (current severity); 10-point scale (psychosocial impact); 10-point scale (historical course and interventions)	0–70	Does not include separate components for different symptoms, includes a single item to assess psychosocial impact of psoriasis	Strong correlation with PASI, adequate reliability, reflects the functional and psychological impact of psoriasis extent
Psoriasis Symptom Inventory (PSI) [94]	Psoriasis sign and symptom severity	Two versions: 7-day and 24 h	Itching, redness, scaling, burning, stinging, cracking, flaking, and pain	4-point scale for each symptom/item	0–32	No published data regarding its responsiveness to deterioration	Adequate reliability, able to detect improvement
Impact of Psoriasis Questionnaire (IPSO) [87,98]	Psychosocial effect of psoriasis	Specified in item: daily, last month	Physical (3), psychological (8), social components (5)	5-Point adjectival scale	0–64	Short recall period	Well-demonstrated reliability; the IPSO-11 Rasch version [87] had sufficient evidence for a strong recommendation for use according to the COSMIN guidelines [84,85,99].
Psoriasis Index of Quality of Life (PSORIQoL) [100]	Psoriasis-related needs-based quality of life	Not reported	1 Scale (25)	True/false	0–25	The yes/no response scale might lack sensitivity, especially with small changes	High test–retest reliability; good psychometric properties
Pictorial Representation of Illness and Self Measure (PRISM) [103]	Self-illness separation score (SIS)	Present time	2 discs, representing the respondent and psoriasis	Positioning of a red disk on a board	0–27 cm	It is difficult to say what PRISM exactly measures, requires a trained administrator, unlikely to be feasible for routine clinical use	Allows a non-verbal definition of global suffering due to illness, demonstrated validity in many studies assessing other diseases

## 4. Future Directions

Regulatory bodies and guidelines now encourage clinicians and researchers to include validated PRO tools to assess psoriasis-related physical, psychological, and social well-being for endpoint analyses in clinical trials and routine care, in addition to objective severity measurement [104,105]. Even so, clinical decisions on systemic treatment in patients with moderate-to-severe psoriasis are still more frequently based on PASI than DLQI [106]. It has been reported that healthcare providers face obstacles in using patient-reported outcomes (PROs) in clinical practice due to concerns about time constraints during consultations and doubts about the usefulness of PROs in guiding treatment plans [39,40]. In addition to representing the patient’s perspective, participating in PRO measures should not be overly difficult, time-consuming, or stressful (so-called respondent burden) [107]; PROs should offer swift, easily understandable scoring and interpretation for clinicians, be able to capture changes resulting from treatment or interventions [38], and reflect patient satisfaction with their treatment [108]. To be truly effective in clinical settings, PRO measures should have validated descriptors for the scores, akin to what is available for certain existing measures such as DLQI, Skindex, and SPI [109]. However, descriptors for most psoriasis-specific PRO measures are not yet available. Furthermore, information about the minimal important clinical difference [46] is also beneficial for clinical interpretation. A variety of specific instruments now exist for measuring quality of life in psoriasis, but as their numbers grow, it becomes more challenging to interpret and use the data collected [110]. Therefore, it is advisable to clearly define the need for developing additional quality-of-life instruments in dermatology before creating any new questionnaires [111]. Rasch analysis [112] is considered the preferred method for developing and enhancing questionnaires, as it offers several advantages over traditional test theory approaches like factor analysis [75,101,113,114,115,116,117]. Although numerous PRO measures are used in psoriasis, only the US English version of the IPSO-11 Rasch questionnaire has recently demonstrated sufficient evidence to be recommended for use [87]. Despite various documented criticisms [62,98] and questions about its usefulness [118], the DLQI maintains its status as a valuable measurement tool in dermatology due to its intentional design for simplicity and ease of interpretation [119].

The highly effective and safe profile of biologics now makes complete “clearance” of psoriasis lesions a realistic goal, facilitating dermatologists’ intervention on psoriasis comorbidities and HRQoL. Several areas of interventions should be addressed in order to improve treatment effectiveness and alleviate psoriasis burden. Severity scores to be considered for reimbursement of treatment with biologic drugs may vary across different countries. For example, in the UK, PASI ≥ 10 and DLQI > 10; in Hungary, PASI > 15 and DLQI > 10; in Poland, PASI > 18 and DLQI > 10; or in Croatia, PASI >15 and/or BSA > 15 and/or DLQI > 15 are required to receive reimbursement [120,121]. It is of note, however, that healthcare providers in several countries do not consider QoL information at the moment for treatment reimbursement purposes, and, in practice, severe psoriasis is considered for PASI > 10 or involvement of “critical” anatomical sites. Harmonization in implementing international guidelines into local reimbursement criteria for systemic therapy that includes PRO measures is desirable. Although psoriasis is associated with such physical and psychological burden, adherence to prescribed treatments is often low, and surprisingly, adherence rates are lowest among patients with the most severe disease [122]. Thus, it is relevant to improve patient adherence to topical therapies [123]. Psychological factors have repeatedly been shown to be associated with non-adherence; particularly addressing depression as a frequent comorbidity may be a currently neglected opportunity to improve care [124]. Similarly, dermatologists should be able to capture possible discrepancies between CRO and depression or other psychological conditions that do not seem to be entirely or clearly traceable to the patient’s psoriasis, addressing psychosocial impact for appropriate counselling [125]. On the other hand, the management of physical comorbidities of psoriasis may also improve HRQoL through interventions on lifestyle. For instance, a low-caloric diet may positively impact the severity of psoriasis and QoL of patients with psoriasis [126]. Patient educational programs were shown to significantly reduce DLQI [127] and PDI [127,128].

## 5. Conclusions

When deciding on systemic treatment for moderate-to-severe psoriasis, it is crucial to consider a broad definition of severity that includes disease burden. Dermatologists should be able to engage in an open dialogue with their patients to understand their needs and expectations regarding their condition and treatment. Consistent validation and enhancement of psoriasis-specific PRO measures should provide healthcare professionals with more accurate tools for effectively and comprehensively assessing the impact of psoriasis in everyday practice in the future. Dermatologists, being able to address the patients’ psychological well-being, can now play an even more crucial role in taking care of psoriatic patients as a whole. Patients with psoriasis are not only meant to be treated but may also become aware of how to improve their QOL thanks to safer, effective medications.

## Data Availability

Data sharing is not applicable to this article.
